# Prolonged Zika Virus RNA Detection in Semen of Immunosuppressed Patient

**DOI:** 10.3201/eid2508.181543

**Published:** 2019-08

**Authors:** Christina Petridou, David Bonsall, Aleem Ahmed, Mark Roberts, Carolyn Bell, Mariateresa de Cesare, Rory Bowden, Victoria Graham, Daniel Bailey, Andrew Simpson, Emma Aarons

**Affiliations:** Rare and Imported Pathogens Laboratory, Public Health England Porton, Salisbury, UK (C. Petridou, V. Graham, D. Bailey, A. Simpson, E. Aarons);; University of Oxford, Oxford, UK (D. Bonsall, M. de Cesare, R. Bowden);; Leicester Royal Infirmary, Leicester, UK (A. Ahmed);; Worcestershire Royal Hospital, Worcester, UK (M. Roberts);; South Warwickshire National Health System Foundation Trust, Warwick, UK (C. Bell)

**Keywords:** Zika virus, semen, persistence, clearance, immunosuppression, viruses, United Kingdom

## Abstract

Zika virus RNA has been detected in semen samples collected <370 days after symptom onset. We report unusual persistence of Zika virus RNA in semen, confirmed by sequencing at 515 days after symptom onset and detectable for >900 days, in a patient with immunosuppression.

Detection of Zika virus RNA in semen was described previously in an immunocompetent man 370 days after symptom onset; envelope and precursor of M protein gene sequencing indicated high genetic stability in semen 3–4 months after symptom onset (*1*). We report detection of Zika virus RNA in semen over a longer period in a 43-year-old immunosuppressed man in the United Kingdom.

The patient has multicentric reticulohistiocytosis (MRH), a rare rheumatologic condition, which was diagnosed in 2015. When MRH was diagnosed, the patient had multiple pruritic, firm papules and nodules on his face and neck. He also had lesions with a characteristic coral bead appearance at periungal sites. In addition, he had severe joint pain and stiffness affecting his hands and knees and drenching sweats. His MRH diagnosis was confirmed by testing of a punch biopsy of a lesion. He was HIV negative, and his immunoglobulin levels and immunoglobulin electrophoresis results were normal. He was initially treated with topical steroids and antihistamines, but he only had limited relief. He was prescribed oral steroids and required high doses to control his symptoms. Clinicians added methotrexate and hydroxychloroquine to his medications as steroid-sparing agents and to reduce the chance his MRH would progress to erosive disease.

In April 2016, seven months after starting his disease-modifying antirheumatic drugs, the patient experienced fever and a new widespread maculopapular rash. He had returned to the United Kingdom from Brazil 7 days before. We detected Zika virus RNA in plasma taken 1 day after symptom onset by using real-time reverse transcription PCR methods described by Pyke et al. (*2*), with modifications (Appendix). We did not detect Zika virus nonstructural protein 1–specific antibodies by ELISA (EUROIMMUN, https://www.euroimmun.com) in initial samples, but we noted seroconversion on day 13 (Appendix Figure). After diagnosing Zika virus infection, clinicians stopped the patient’s methotrexate and hydroxychloroquine, but he remained on prednisolone to prevent a flare-up of his MRH (Appendix Figure). The patient’s clinical course of Zika virus infection was unremarkable.

A previous study reported that Zika virus RNA was detected in several semen samples taken within 6 months of symptom onset (*3*). We tested subsequent semen samples from this patient and found Zika virus RNA persisted at a viral load sufficient for sequencing 515 days after symptom onset (Table; Appendix Figure). To date, Zika virus RNA remains detectable in further semen samples, although at higher cycle threshold values (Table). The patient remains asymptomatic for Zika virus infection despite persistent detection of Zika virus RNA in his semen.

**Table:** Serial Zika virus genome sequence and culture results from semen of a patient with immunosuppression, United Kingdom*

Sample no.	Days after symptom onset	RT-PCR C_t_ value†	Sequence coverage, %‡	Average read depth	Sequencing platform§	Mutations detected	Culture result
1	13	19	99.9 (min depth 2), 88.4 (min depth 40)	386.9	MinION	Reference	Frozen sample: unsuccessful
2	46	26	ND		NA	NA	Fresh sample: unsuccessful
3	167	Subthreshold	ND		NA	NA	ND
4	194	No RNA detected¶	ND		NA	NA	ND
5	241	31	Unsuccessful		NA	NA	ND
6	257	34	44 (min depth 2), 0 (min depth 40)	3.3	MinION	None	Frozen sample: unsuccessful
7	278	No RNA detected¶	ND		NA	NA	ND
8	326	26	76 (min depth 2), 6 (min depth 40)	13.2	MinION	None	Fresh sample: unsuccessful
9	396	29	ND		NA	NA	ND
10	515	24	98.1 (min depth 5)	33.3	MiSeq	K3272E, Syn2921	Fresh sample: unsuccessful
11	687	39	ND		NA	NA	ND
12	941	32	ND		NA	NA	ND

*C_t_, cycle threshold; min, minimum; NA, not applicable; ND, not done; RT-PCR, reverse transcription PCR. †Before PCR, nucleic acid was extracted from samples 1–7 using the EZ1 Virus Mini Kit (QIAGEN, https://www.qiagen.com). Samples 8–12 were extracted using the MagNA Pure 96 DNA and Viral NA Small Volume Kit (LifeScience-Roche Diagnostics Corporation, https://lifescience.roche.com). C_t_ values <40 with acceptable amplification curves are interpreted as positive, but results for samples with C_t_ values >35 are confirmed by reextraction and repeat PCR in triplicate, where possible. ‡Conservative read-depth thresholds were selected for comparative analyses of the day 13 (sample 1) and day 515 (sample 10) consensus genomes. §MinION (Oxford Nanopore Technologies, https://nanoporetech.com); MiSeq (Illumina, https://www.illumina.com). ¶Confirmed on reextraction and repeat PCR testing.

We attempted viral culture on multiple semen samples, as previously described (*3*), but were unsuccessful (Table). We constructed sequencing libraries from total seminal plasma-extracted RNA enriched by using a panel of oligonucleotide probes, 120 nt in length, designed to capture all known Asian Zika virus strains, according to previously described methods (*4*). We prepared libraries for previously collected semen samples from before day 326 and sequenced these using MinION (Oxford Nanopore Technologies, https://nanoporetech.com). We did the same for the day 515 sample and sequenced it using MiSeq (Illumina, https://www.illumina.com). We used double indexing to prevent cross-contamination and index misassignment errors. 

We also prepared 90 plasma samples from patients infected with hepatitis C, collected for a separate study, in parallel with the day 515 sample. We did this to exclude the possibility of cross-contamination from our patient’s previous samples, particularly the day 13 sample, which were shipped, prepared, and sequenced 6 months earlier. We did not detect Zika virus in any of the hepatitis C samples. We found no evidence of cross-contamination with Zika virus sequences during processing that could explain the near-whole genomes detected in the day 515 sample. Consensus sequences were consistent with all samples having come from the same patient with only 2 mutations, 1 synonymous change at codon 2921 and a K3272E substitution, acquired during the 502 days between the first and last samples sequenced (Table). We deposited sequence data in GenBank (accession nos. MH763832–3).

Counotte et al. systematically reviewed all available evidence on the risk for sexual transmission of Zika virus (*5*). Data from case reports, case series, cohort studies, in vitro work, and animal studies indicate that the infectious period for sexual transmission of Zika virus is considerably shorter than the period during which viral RNA can be detected in semen. As a result, the World Health Organization now recommends male travelers with potential Zika virus exposure delay conception for >3 months rather than >6 months (*6*). 

In our case, Zika virus RNA might have persisted in semen because of failed immune clearance secondary to the patient’s MRH or his immunosuppressive drug treatment. However, when advising returning male travelers in couples planning pregnancy, clinicians should be aware that Zika virus RNA shedding in semen might be intermittent and persist for longer in patients with immunosuppression.

Appendix Additional information for study of prolonged Zika virus RNA detection in semen of patient with immunosuppression.
