# Supplementary material for: Prolonged Zika Virus RNA Detection in Semen of Immunosuppressed Patient
Source: Emerg Infect Dis. 2019 Aug;25(8):1598–600. doi: 10.3201/eid2508.181543 (PMC6649331; doi:10.3201/eid2508.181543)
Supplement: Appendix — Additional information for study of prolonged Zika virus RNA detection in semen of patient with immunosuppression. [file 18-1543-Techapp-s1.pdf]

Article DOI: https://doi.org/10.3201/eid2508.181543

# Prolonged Zika Virus RNA Detection in Semen of Immunosuppressed Patient

## Appendix

### Modifications to real-time reverse transcription PCR methods for detecting Zika virus RNA

We used real-time reverse transcription PCR methods described by Pyke et al. (1) with the following modifications: we incorporated MS2 as an internal control; made slight changes to the forward primer and added a second probe to better cover African strains; and we replaced of the 3’ TAMRA dye on the probes with BHQ1.

#### Reference

1. Pyke AT, Daly MT, Cameron JN, Moore PR, Taylor CT, Hewitson GR, et al. Imported Zika virus infection from the Cook Islands into Australia, 2014. PLoS Curr. 2014;6:1–7.

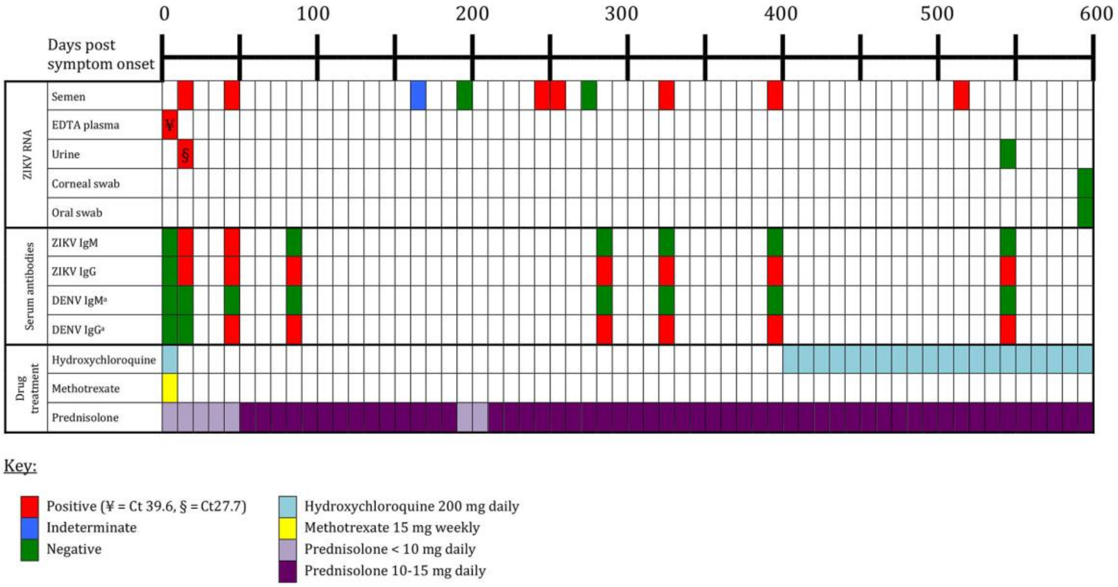


**Appendix Figure.** Clinical case timeline of PCR and serology investigations, and drug treatment for patient with immunosuppression and prolonged presence of Zika virus RNA in his semen.
